# Synthesis and biodegradation testing of some synthetic oils based on ester

**DOI:** 10.1038/s41598-024-53331-6

**Published:** 2024-02-10

**Authors:** Reham I. El Shazly, Huda S. El-Sheshtawy, Nehal S. Ahmed, Amal M. Nassar

**Affiliations:** 1 Department of Petroleum Applications, Egyptian Petroleum Research Institute, Nasr City, 11727 Cairo Egypt; 2 Department of Biotechnology, Egyptian Petroleum Research Institute, Nasr City, 11727 Cairo Egypt

**Keywords:** Environmental sciences, Chemistry, Engineering, Materials science

## Abstract

Synthetic ester oils are widely used in many applications due to their ideal cleaning properties, lubricating performance and assured polarity. The majority of esters oils are more biodegradable. than any other base stock. For instance, oil soluble polyalkyleneglycols (PAGs) or polyalphaolephins (PAOs), are only biodegradable in the lower viscosity grades. The goal of this study is to create some synthetic base oils by two major protocols; the first is esterifying valeric acid with various glycols (ethylene glycol, propylene glycol, butylene glycol and poly (ethylene glycol 400). The second involves esterification of propanoic acid, heptanoic acid, or octanoic acid with ethylene glycol. The reaction yield varies between 85 and 94%. The chemical composition of the prepared esters was examined using various spectroscopic methods (*Fourier-transform infrared* (FT-IR) and *proton nuclear magnetic resonance* (^1^H-NMR) spectroscopy. The thermal properties investigation by *thermo gravimetric analysis* (TGA) showed pronounced thermal stability of the prepared esters. The biodegradability was verified versus two bacterial isolates (B1, B2). The results showed that percentage of degradation of the lube oil was in the range of 34% to 84% after 3 days of incubation. Moreover, the rheological study revealed that the prepared esters exhibited *Newtonian* rheological behaviours. Viscosity examination displayed that the esters based on ethylene glycol, such as (A), had the highest VI: 179 values when compared to those based on higher glycols. Viscosity and viscosity index results showed slight increase as the number of carbon atoms in the acid chain increases. At last, most of the synthesized esters possessed pour points ≤ − 32 °C: ≤ − 40 except in case of using higher acids like heptanoic acid and octanoic acid in preparation the pour point increases to − 9 °C and − 15 °C.

## Introduction

Various microorganisms have the ability of utilizing hydrocarbons as their sole carbon source for metabolic activity, such microorganisms are plentiful in nature. The hydrocarbon used by each microbic strain is dependent on the chemical composition of the petroleum mixture, as well as other environmental considerations^1^. One of the common sources of the hydrocarbons is Lubricating oil which represents a widespread pollutant in both water and soil. Lubricating oil typically contains 80% hydrocarbon base oil and the rest is different additives. On the other hand, the green chemistry approach emphasizes the use of environmentally friendly materials- as possible- in different industries and applications^2^. This trend of replacing petroleum-based oils with more environmentally friendly insulating oils can also be seen in oil field transformer problems. Lubricant is emitted into the environment in the form of oil vapor and viscous micro droplets, creating a severe environmental risk. The power and impact of oil derivative interactions are directly related to the content, volume, and frequency of emissions in a particular area, as well as the attributes with open cutting system device [ref]. As known, mineral oils have a very low biodegradability [ref]. Also, Petroleum-derived oil poses significant risks to sawing operators in the natural environment. On the other hand, oil dangerously accumulates in different parts of plant, animal tissues, and even in groundwater causes various risks. More specifically, crude oil derived lubricants are a major danger to aquatic habitats even at very low concentrations^3^. However, Synthetic or vegetable oils are considered more suitable substitutes^4^. When designing the lubricant content, both environmental and application parameters must be taken into concern. As a result, it must be distinguished not just by a specific rate of biodegradability, but also by suitable physicochemical aspects^3^, such as the viscosity index range, dynamic and kinematic viscosities at different temperatures, pour point, evaporability, flash points, and the base or acid number. The use of appropriate (biodegradable) insulating oils would significantly improve the environmental aspects of all power transformer operations. This premise serves as the foundation for the primary intent of this paper, which is to investigate the possibilities of substituting suitable synthetic or natural products for petroleum-based oils^4^.

Transesterification and esterification reactions are commonly performed in hydrocarbon solvents such as toluene. The most common homogeneous acid catalysts for this purpose are *sulfuric acid*, *methane sulfonic acid*, and *p-toluene sulfonic acid*^5,6^. The usage of these catalysts poses a few obstacles, such as corrosion beside their environmental hazards. As a result, new methodologies are being developed involving more efficient catalysts that do not require solvent^7,8^. It is generally known that heterogeneous catalysts are used in liquid phase organic reactions, which can provide numerous advantages. After simple filtration, the catalyst is easily recovered, and the reaction product solution is clean. Therefore, some considerations have been taken into account in esterification processes to the employment of heterogeneous catalysts^2^. On the other hand, bioremediation is a type of biodegradation in which microorganisms are used to free the environment from organic and inorganic xenobiotics via their enzymatic activity^9^. Indeed, bacteria and fungi degrading hydrocarbons are abundant in marine, freshwater, and soil habitats^10^.

This research focuses on synthesis of *diol diesters* using *Amberlyst®15* heterogenous catalyst by interaction of different *dihydric alcohols* (*ethylene glycol*, *propylene glycol*, *butylene glycol*, and *poly ethylene glycol 400*) with different *monoacids* (*propanoic, heptanoic, octanoic*, and *valeric acids*) and evaluating them to be used as highly performed synthetic lubricants and measuring the rate of biodegradability of these esters using two types of microorganisms.

## Experimental

### Materials

*Acids (propanoic, heptanoic, octanoic, and valeric acids)* were acquired from *Aldrich Chemical Co. Ltd. (UK). Ethylene glycol (EG), propylene glycol (PG), and butylene glycol (BG)* were acquired from *VEB LABORCHEMIE APOLDA*. *Polyethylene glycol 400 (PEG 400)* was maintained from *Morgan Chemical Ind. Co.* Other chemical reagents and organic solvents were of analytical quality and acquired from *Carlo Erba Reagenti*. *Amberlyst 15* used came from Alfa Aesar Co. with CAS no. 6192-52-5.

### Procedure

#### Esterification reaction

The preparation of three diesters was achieved by a facile methodology. Simply, 2 mol of *valeric acid* with one mole of different *glycols* (*ethylene glycol, propylene glycol, butylene glycol, and polyethylene glycol 400*) were placed inside a closed 1L. three-necked flask fitted with a Dean-Stark trap (filled with xylene). Also, the other three *diol glycols* were prepared by reacting two moles of different *acids (propanoic, heptanoic, or octanoic acid*) with one mole of *ethylene glycol* in the same manner in presence of *Amberlyst®15* (0.5 wt%) as a catalyst the reaction was held in *xylene*. Slow flow of *deoxygenated nitrogen* 40 ml/min. was applied to avoid oxidation. The reactants with the same weight of *xylene* as a solvent are gradually heated at a controlled thermostat temperature up to 120 °C ± 0.5 °C and stirred at 500 rpm^11^. The reaction was held for 8 h to reach a product yield of 83–92%. To find out the rate of the reaction, the amount of freed water in Dean-Stark trap was measured.

#### Purification of prepared esters

A 10% by weight aqueous solution of *sodium carbonate* was used to wash the organic layer in the separating funnel, followed by multiple washing with distilled water. To remove *xylene*, under vacuum, a rotary evaporator was utilized. Then the products were dryed by using *sodium sulfate anhydrous* overnight^12^. The codes and composition of prepared esters are given in Table 1.

**Table 1:** The designation of prepared esters.

Designation	Prepared dibasic ester composition
A	2 mol. *Valeric acid* + 1 mol. *ethylene glycol*
B	2 mol. *Valeric acid* + 1 mol. *propylene glycol*
C	2 mol. *Valeric acid* + 1 mol. *butylene glycol*
D	2 mol. *Valeric acid* + 1 mol. *poly ethylene glycol 400*
E	2 mol. *Propanoic acid* + 1 mol. *ethylene glycol*
F	2 mol. *Heptanoic acid* + 1 mol. *ethylene glycol*
G	2 mol. *Octanoic acid* + 1 mol. *ethylene glycol*

### Characterization of prepared compounds:

Table 2 displays the analysis used to identify the chemical composition and examine the physicochemical properties of the prepared di*esters*.

**Table 2:** Analysis to confirm the chemical structure and the physicochemical properties of prepared di*esters.*

Analysis	Apparatus/ specifications	Test method
*Infra-red spectroscopic analysis*	*Fourier Transform Infrared Spectroscopy* (FT-IR.) Spectrometer Model Mattson- Infinity series Bench top 961	
^*1*^*H-NMR spectroscopic analysis*	300 MHz spectrometer W.P. 300, Bruker using DMSO as a solvent	
*Thermogravimetric analysis* (TGA)	Evaporation loss of lubricating oil by TGA 55 Rate: 20 °C min^−1^ Temperature: 600 °C Under: Nitrogen gas	ASTM D 6375-99A
*Density*	Density at 15 °C (g/ml)	ASTM D 1298
*Flash Point*	Cleveland open cup tester	ASTM D 92
*Viscosity Index*		ASTM D 22–70
*Pour Point*	Seta cloud and pour point cryostat	ASTM-D 97
*Rheological and Tribological Properties*	Model type the modular compact rheometer 502 (Anton Paar)	

### Biodegradation measurements

#### Isolation of hydrocarbon utilizing bacteria

Oil degrading bacteria have been isolated via *mineral salts medium (MSM*). The *MSM* (g/L) includes the following ingredients: *0.5 MgSO*_*4*_*·7H*_*2*_*O; 0.5 KH*_*2*_*PO*_*4*_*; 0.1 KCl; 4.0 NH*_*4*_*NO*_*3*_*; 1.0 K*_*2*_*HPO*_*4*_*; 2.0 NaCl; 0.01 CaCl*_*2*_*; and 0.01 FeSO*_*4*_*·7H*_*2*_*O.* The pH of the media was adjusted to 7.0 ± 0.2 by using 1% oil as the sole carbon source. The 250 mL conical flasks were inoculated with 100 mL of *MSM* broth The inoculated sample flasks were incubated for 7 days at 30 ± 2 °C in a shaking incubator at 150 rpm^13^. The bacterium colonies that grew on the plates were chosen for further investigation.

#### Selection of the most promising bacterial isolates

The most promising bacterial isolates for oil degradation (B1 and B2) grew faster on *MSM* mixed with oil as the main carbon source. They were chosen for further research.

#### Molecular identification of the bacterial isolate

The degrading bacterial isolates (B1 and B2) were identified in *Sigma Scientific Services Co.*, Egypt, using 16s *rDNA*. After *PCR* product purification, the positive clone's *DNA* sequence was compared by the use of BLAST on the NCBI website (http://www.ncbi.nlm.nih.gov) and deposited in GenBank. Many relevant 16S *rDNA gene* sequences with currently published names were picked from the Gen-Bank as references.

#### Screening for the ability of bacterial isolates to degrade lubricating oil

By the use of *mineral salt medium (MSM*), the bacterial isolates were tested because of their capability of using lubricating oil as a main carbon source. Individual bacterial Cells were introduced into 250 ml Erlenmeyer conical flasks in amounts of 5 ml. Each containing 100 ml of *sterile MSM* containing 1% v/v of five different lubricants. The experiment was done in triplicate, with uninoculated flasks serving as controls. The flasks were all incubated at 30 °C for 72 h at 150 rpm and pH 7.5. The leftover crude oil samples have been taken out from various cultural microcosms and analyzed gravimetrically and chromatographically.^14^.

#### Analysis of lubricating oil biodegradation

Each flask's remaining oil was extracted using the *US-EPA Gravimetric Method* (1999) after the predetermined incubation period. In a nutshell, an oil-containing sample was gathered in a 500-mL separating funnel. By adding 6 N *HCl*, the pH was reduced to 2. *Carbon tetrachloride* was used twice to extract the oil in the samples. The solution's remaining solvent was then evolved in a rotary evaporator. Finally, the concentrated oil flask was placed in a desiccator for drying. Gravimetric analysis was used to determine the amount of residual oil recovered^15^. The percentage of oil content reduction was calculated as (A − B)/A × 100, where A is the initial crude oil weight and B is the remaining crude oil weight.

#### Determination of normal and iso-paraffins

The residual hydrocarbons were quantified chromatographically with *Agilent 6890 plus capillary gas chromatography (GC)*^16^. The peak area of each resolved constituent (whether *n- or iso-paraffins*) was measured separately. While the *UCMs* were made up of *non-n-paraffins*, primarily *cycloparaffins* and *aromatics* with long side chains, they were only detected as a total hump.

## Results and discussion

There is an urgent need to develop pollution-free and environmentally friendly lubricants as an alternative to mineral-based lubricants^17^. Rape, soybean, and palm oils are among examples of basic oils. They possess good tribological properties. Despite their obvious benefits, they have limited corrosion resistance and are in danger of hydrolytic and oxidative instability^18^. *Esters of glycols* are an effective replacement for mineral and vegetable oils. This research focuses on the synthesis of *diesters*, the rate of biodegradability of the prepared *esters*, and their suitability for use as synthetic lubricants.

### Preparation of diesters

**Figure 1:**
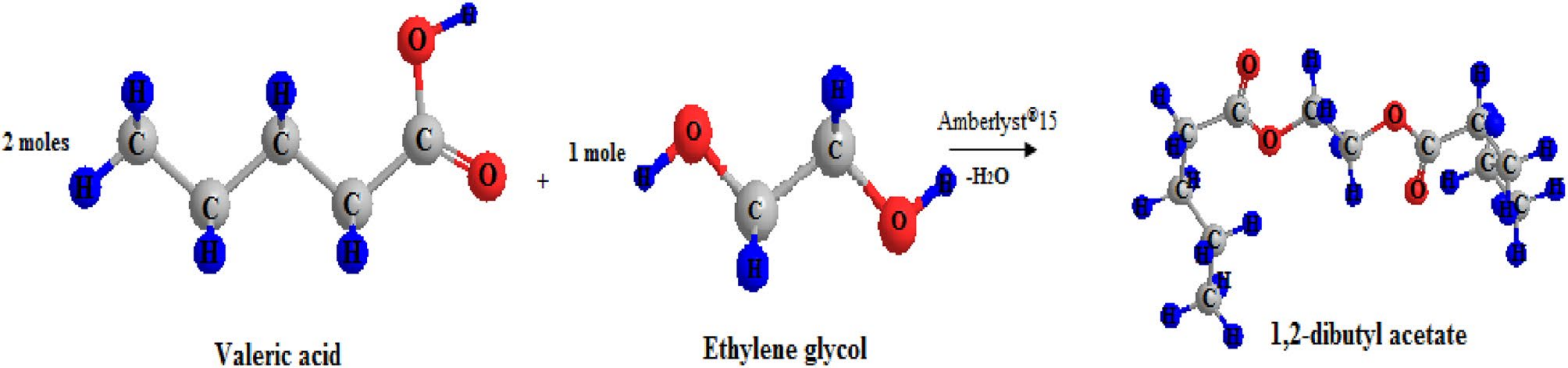
The esterification reaction of *valeric acid* and *ethylene glycol*.

*Diesters* were produced by individually reacting *valeric acid* with various *glycols (ethylene glycol, propylene glycol, butylene glycol, or polyethylene glycol 400*). Then, *ethylene glycol* reacts with lower (*propanoic acid*) and higher (*heptanoic or octanoic acid*) acids separately. The overall esterification reaction is shown in Fig. 1.

### Structure confirmation of the prepared esters

#### FTIR

**Figure 2:**
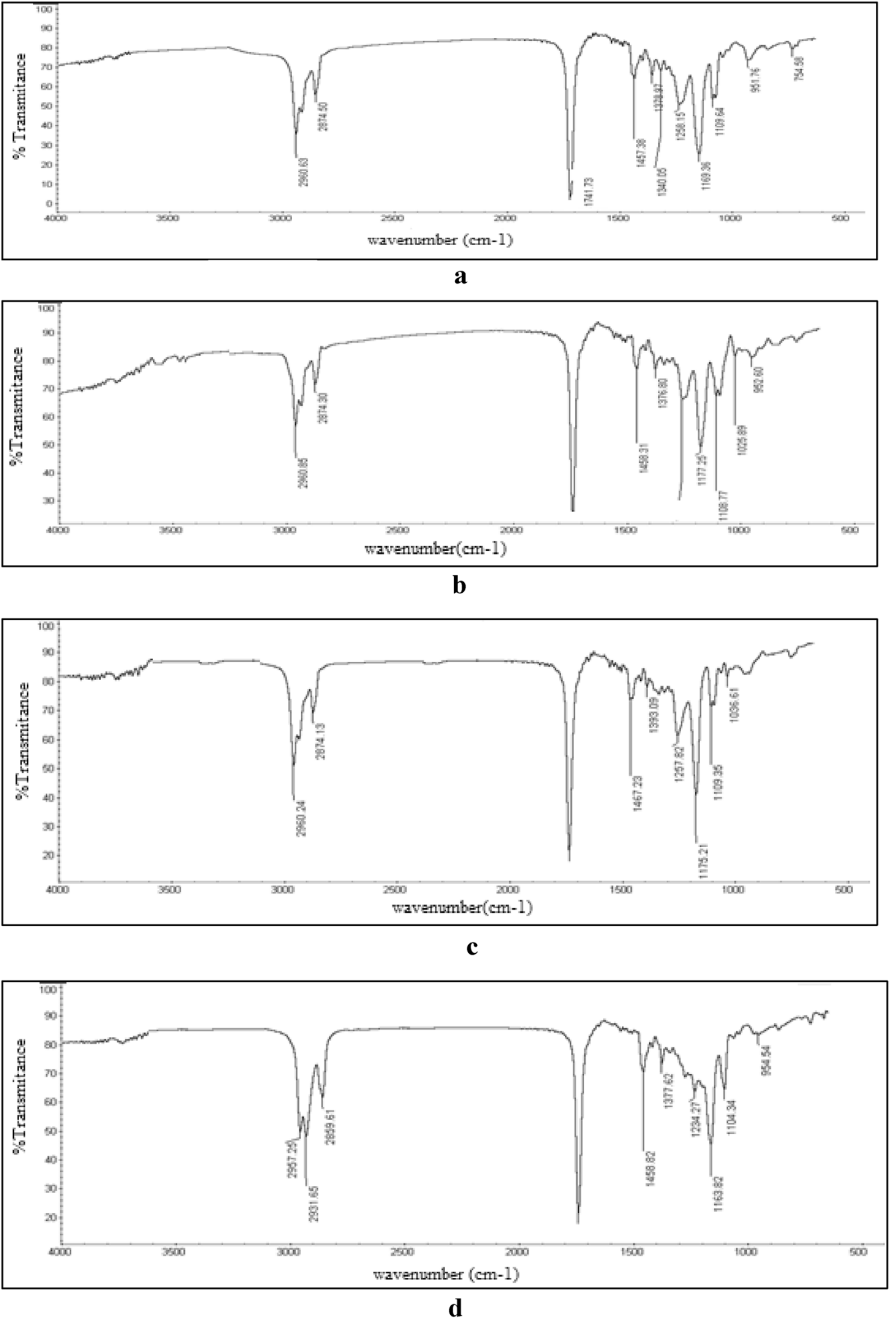
*IR* spectrum of compound A (**a**), compound B (**b**), compound C (**c**), compound F (**d**) .

FTIR spectroscopy was used to ensure the completion of esterification reactions. The spectra of all *esters* are nearly the same with slight differences as seen in Figs. 2a–d. Generally, the FTIR spectra display the following: the disappearance of strong peaks –OH at 3500 cm^−1^ ± 10 cm^−1^ and *acid* group band C=O at 1730 cm^−1^ at the end of esterification and their replacement by *ester* group bands C=O at 1735 cm^−1^ and 1265 cm^−1^ ± 10 cm^−1^, respectively. This demonstrates that the esterification procedure was carried out successfully^11^.

#### ^1^H-NMR spectroscopy

**Figure 3:**
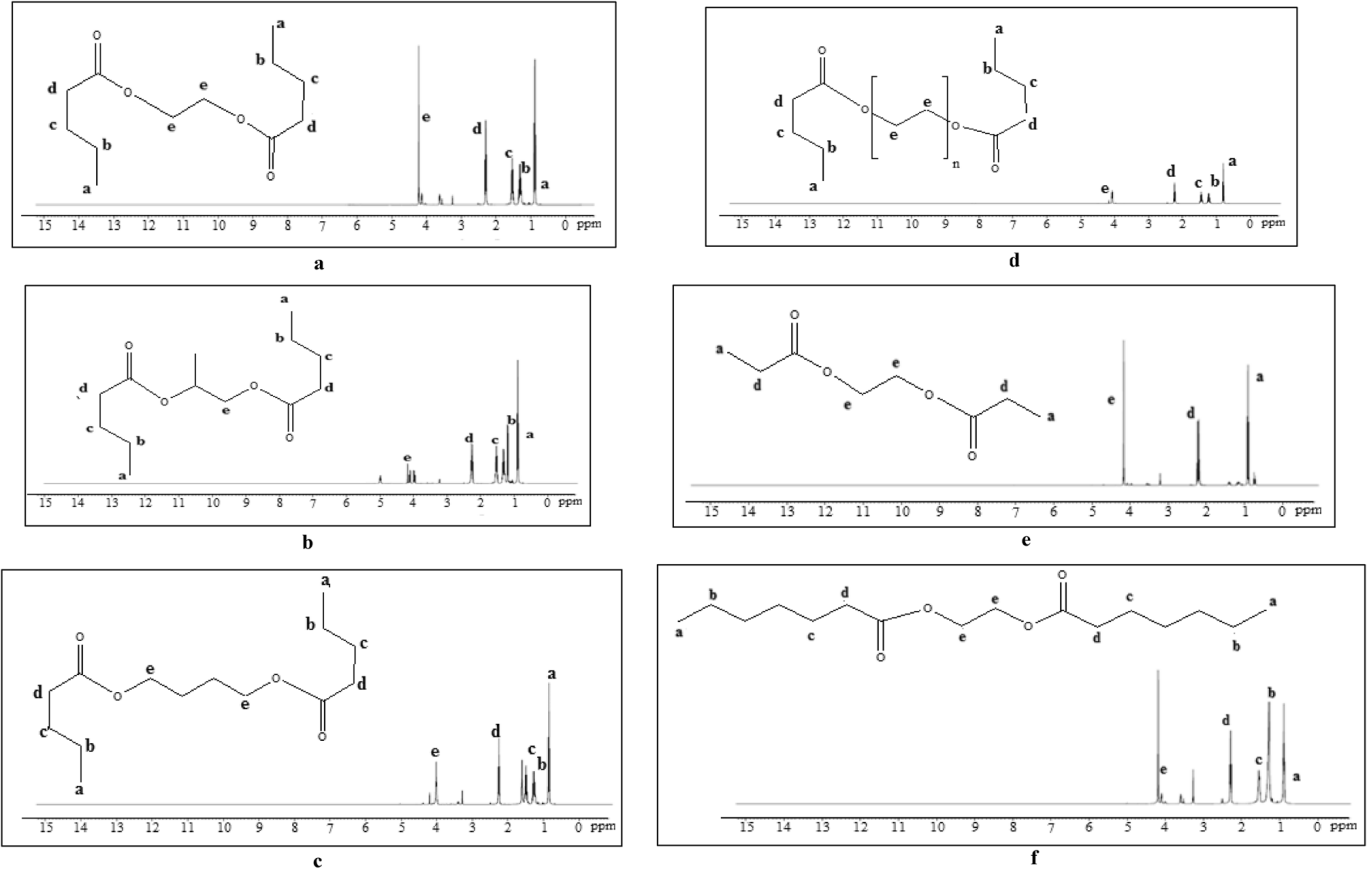
The ^*1*^*H-NMR* spectrum of compound A (**a**), compound B (**b**), compound C (**c**), compound D (**d**), compound E (**e**), compound F (**f**) .

^*1*^*H-NMR* spectroscopy was used to investigate the composition of the products A-F. Figures 3a–f demonstrate *diesters'* chemical shift as follows: ^1^H-CH_3_, triplet at 0.96 ppm (**a**), ^1^H-CH_2_, multiplet at 1.33 ppm (**b**), ^1^H-C–C(O)OR *esters*, multiplet at 1.68 ppm (**c**), ^1^H-C(O)-OC, triplet at 2.25 ppm (**d**), ^1^H-O-C(O)R, triplet at 4.36 ppm (**e**), as well as the loss of RCOOH *carboxylic acid*, which approve the complete acids esterification.

#### Thermal stability of prepared esters

**Figure 4:**
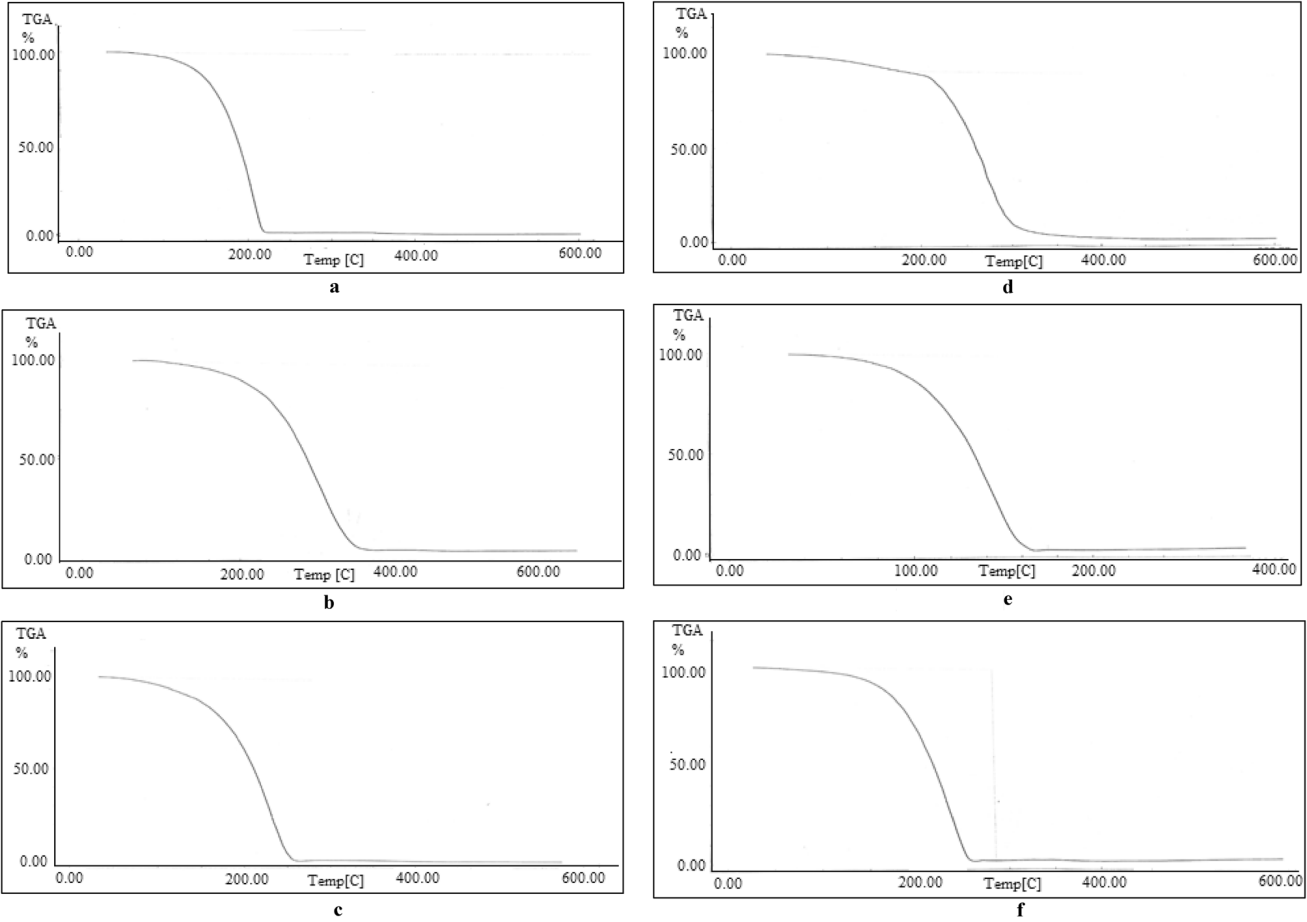
*TGA* of prepared ester A (**a**), ester B (**b**), ester C (**c**), ester D (**d**), ester E (**e**), ester F (**f**) .

The *diesters*’ (A–G) thermal properties were also investigated. For example in Fig. 4a–f the complete degradation occurs between 150 and 300 °C, demonstrating a reasonable thermal stability of prepared *esters*. So, their use is consistent with the temperature restrictions depicted in the thermograms.

### Physicochemical properties of prepared esters

#### Rheological behavior of prepared *esters*

**Figure 5:**
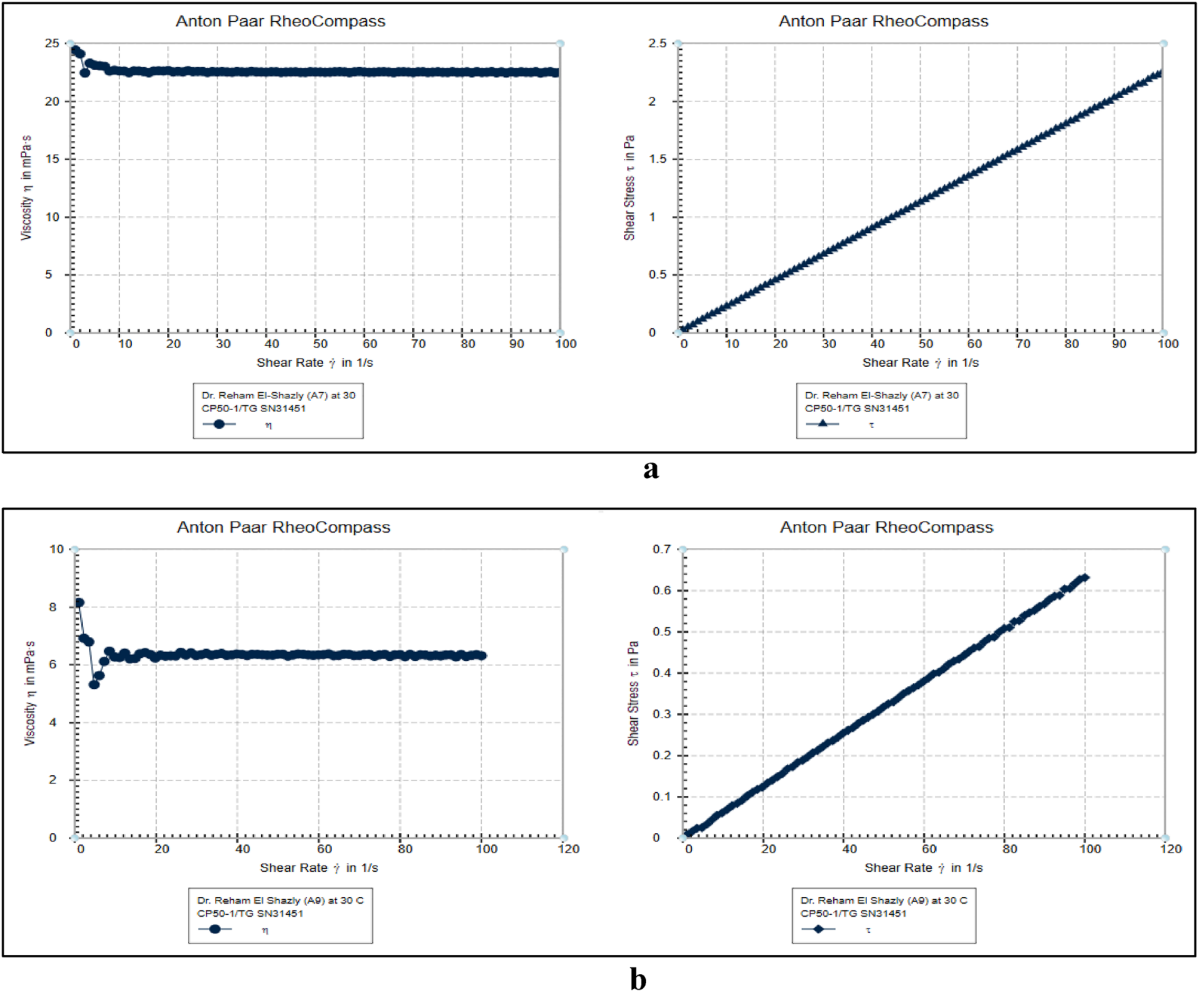
*Rheological behavior* of synthetic oil D (**a**), synthetic oil G (**b**) .

The synthesized compounds have *Newtonian rheological properties*, as illustrated in Fig. 5a,b. This means that *Newtonian fluids* follow *Newton's law* of viscosity. Viscosity is unaffected by *shear rate*. In lubrication, Newtonian fluids like motor oil, are utilized for lubrication purposes in engines and machinery to reduce friction, dissipate heat and protect against wear and tear. Newtonian lubricant is preferable for improving tribological performance. Newtonian fluid can significantly increase the load-carrying capacity of bearing^19^.

### Biological degradability of prepared esters

Culture media (*MSM*) detected seventeen bacterium isolates (B1-B17) from a sample of oil-contaminated water. On *MSM* media, the bacterium isolates B1 and B2 are considered predominant.^15^ determined two biodegrader bacterium species as having the best rate of growth on *MSM* medium.

#### Molecular identification of the selected oil degrading bacteria

**Figure 6:**
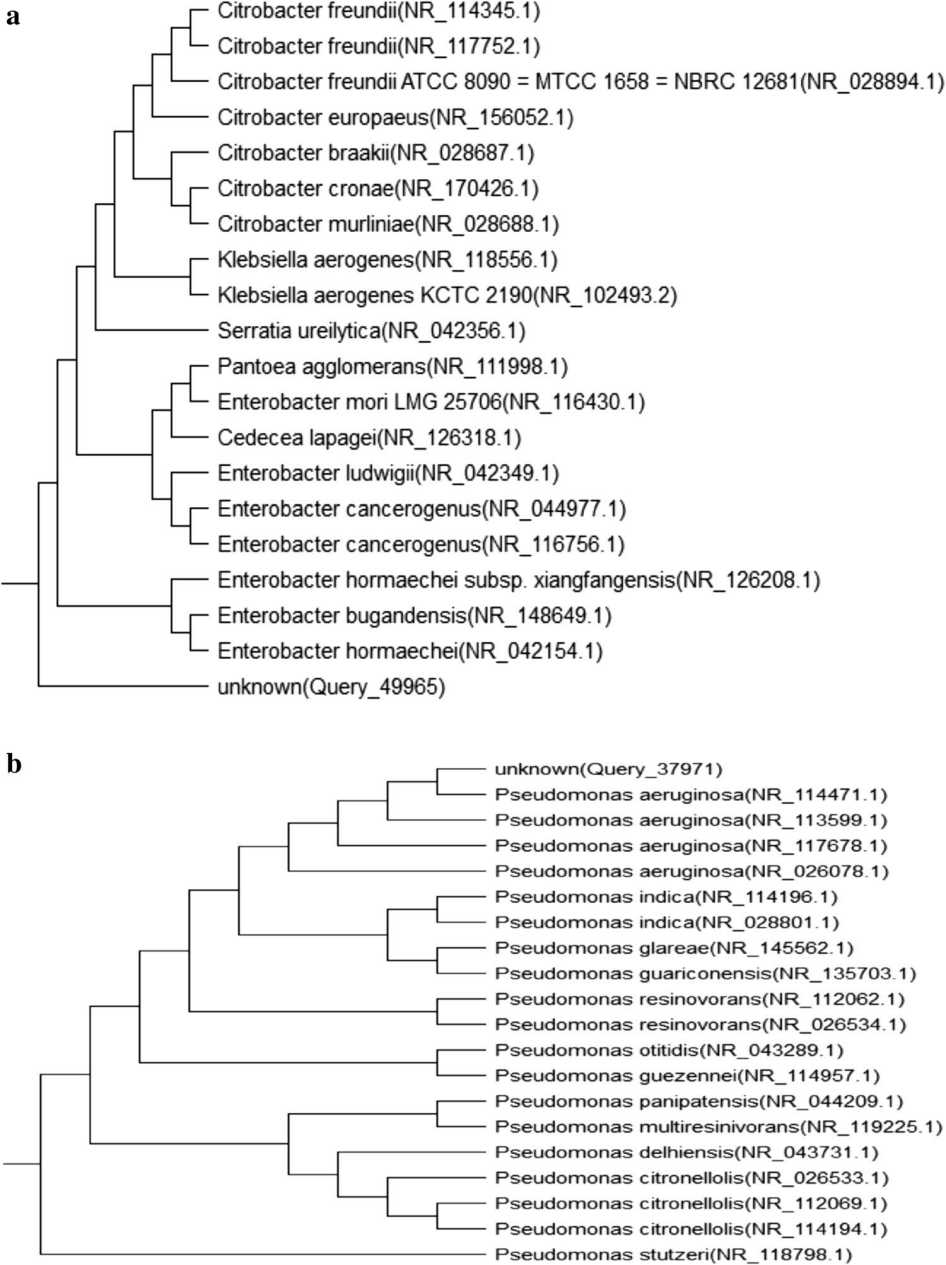
(**a**) A phylogenetic tree of *Enterobacter hormaechei* subsp. Xiangfangensis strain 10–17 16S evolutionary relationships to other strains, taken from the NCBI database and the phylogenetic tree done by the Mega X program. (**b**) A phylogenetic tree of *Pseudomonas aeruginosa* ATCC 10145 evolutionary relationships to other strains, taken from the NCBI database and the phylogenetic tree done by the Mega X program.

Under the light microscope, the bacterium isolate (B1) is a Gram-negative, rod-shaped organism. 16S *rRNA* identifies the bacterial isolate (B2) as *Enterobacter hormaechei subsp. Xiangfangensis strain 10–17* having a 99.17% similarity. MEGA programme is being used. A sequence of Enterobacter hormaechei subsp. identified in this investigation matched with other Enterobacter species. Figure 6a depicts the phylogenetic tree built from Enterobacter hormaechei and closely related bacterial strains using the 16S *rRNA* gene's Neighbor-joining method.

Under a light microscope, the bacterium isolate (B2) appears as a rod-shaped, Gram-negative organism. *Pseudomonas aeruginosa ATCC 10145* is recognised as the bacterial isolate (B2) by 16S *rRNA* with a similarity of 99.53%. using the MEGA programme. Pseudomonas aeruginosa sequences from this study's isolation were matched with Pseudomonas species via BLAST analysis. Figure 6b shows how the phylogenetic tree of *Pseudomonas aeruginosa* and closely related bacterial strains was reconstructed using the Neighbor-joining method of the 16S *rRNA* gene.

#### Biodegradation of different lubricating oil samples using the degrading bacterial strains

In the current study, the capacity of two different bacterial isolates (B1–B2) to biodegrade of the lubricating oil sample was evaluated. The pure bacterial isolates were inoculated into *MSM* media with different lubricating oil separately used as sole carbon source for 3 days. The oil samples were accurately weighted as a gravimetric analysis. The percentage of the oil that had biodegraded was estimated, and *gravimetric analysis (GC)* was used to find the change in chemical composition.

##### *Gravimetric analysis* of the different degraded lubricating oil

According to the results of *gravimetric analysis* in Table.3, different bacterial isolates (B1-B2) degraded lubricating oil in a range from 34 to 84% after incubation for three days. In comparison to other types of lubricating oil, the microcosms containing the individual bacterial isolates (B1 and B2) with A and D gave a higher percentage of degradation. Verma et al.^20^ stated that, after 5 days, Pseudomonas sp. SV 17's oily sludge degradation capacity represents around 60% of the saturates and aromatics components. El-Sheshtawy et al.^16^ demonstrated that after 7 days, the bacterial strains had broken down between 30 and 50% of the crude oil.

**Table 3:** Residual of lubricating oil after biodegradation by two different bacterial isolates.

Lubricating oil	Crude concentration g/l	Bacterial isolate	Weight of crude (residual)	Percentage of degradation%
A	10	B1	2.8	72
B			6.6	34
C			3.7	37
D			4.2	58
G			5.7	43
A	10	B2	1.6	84
B			6.3	37
C			3.5	65
D			3.3	67
G			6.0	40

##### Gas chromatographic analysis

The first step in the mechanism of *alkane* degradation in lubricating oil by bacteria in aerobic conditions is the oxidation of *alkanes* by the class of oxygenase enzymes (enzymes that catalyze the incorporation of oxygen into the substrate) namely *alkane hydroxylase enzymes* that catalyze the addition of *hydroxyl* groups by attacking the O atom through oxidation during the alkane hydroxylation reaction. *Alkanes* are oxidized to *alcohol* and subsequently become *fatty acid*s^21^. The next pathway of *fatty acid* metabolism can be through cellular lipid pathways, β-oxidation, and α-oxidation. Through the β-oxidation pathway *fatty acids* will be converted into *acetyl* CO-A and enter into the TCA cycle, converted into CO_2_ and energy. If through the *fatty acid* α oxidation pathway it will be converted directly into CO_2_ and fat derivatives^22^.

In this study, the biodegradation of various lubricating oils was examined in the current study utilising the *GC* for aliphatic compounds after three days of the incubation period. Following biodegradation by two different bacterial strains, the residual *n-paraffin* and *iso-paraffin* percentages contained in various lubricating oils were calculated and contrasted with the control sample of Tables 4, 5.

**Table 4:** Percentages of residual of *n-* and *iso-paraffins* samples after bacterial degradation by bacterial strain (B1) using *GC chromatography.*

Different lubricating oil		Total paraffins	
		Iso-paraffins	N-paraffins
Control	(A)	2.25	97.75
Sample		0.62	99.38
Control	(B)	5.45	94.55
Sample		3.92	96.08
Control	(C)	1.25	98.75
Sample		1.42	98.58
Control	(D)	5.95	94.05
Sample		3.91	96.09
Control	(G)	1.35	98.65
Sample		1.68	98.32

**Table 5:** Percentages of residual of *n-* and *iso-paraffins* samples after bacterial degradation by bacterial strain (B2) using *GC chromatography.*

Different lubricating oil		Total paraffins	
		Iso-Paraffins	N-Paraffins
Control	(A)	2.25	97.75
Sample		1.06	98.94
Control	(B)	5.45	94.55
Sample		5.02	94.98
Control	(C)	1.25	98.75
Sample		1.36	98.64
Control	(D)	5.95	94.05
Sample		4.52	95.48
Control	(G)	1.35	98.65
Sample		1.16	98.84

The results demonstrated that iso-paraffins degraded faster than n-paraffins, which consider more resistant for the biodegradation process in the most of different microcosms separately. In the literature study The hydrocarbons vary in their sensitivity to microbial attack, and they were previously classed in the following order of decreasing sensitivity: n-alkanes ˃ branched alkanes ˃ aromatics with low molecular weight cycloalkanes^6^. On the other hand, additional high molecular weight substances consumed can be blamed for an increase in any compound's percentage, which causes a proportional buildup of that hydrocarbon component on the low molecular weight chemicals. The data above indicate that (A and D) types of lubricating oil were more consistently degraded by two separate bacterial strains than other types of lubricating oil.

## Evaluation of the prepared esters

A lubricant's primary characteristic is that it is anticipated to lubricate. The lubricity of a molecule refers to how well it coats the metal surface, competes for it, and flows over itself. Because they get in contact with metal surfaces and minimise the amount of metal-to-metal contact during sliding motion, *esters* are typically regarded as effective border lubricants. The chain length, degree of branching, and position of links inside the molecule are structural elements that affect lubricity. Due to oxygen's electronegativity, there is a permanent dipole in the *ester* chemical reaction. This inherent polarity has a number of effects and contributes significant lubricating properties. Permanent dipoles have greater internal cohesion than pure hydrocarbons and are attracted to one another by electrical power, which reduces their evaporation rate and volatility.

If the viscosity is too high, it could result in low oil flow, causing oil starvation and dry startups. When the viscosity is too low, greater mechanical friction may occur, which can lead to heavier loads. You also would see more wear due to film loss.

A higher viscosity index number means the lubricant changes viscosity at a lower rate based on the temperature. A higher VI is more desirable because it enables the lubricant to provide a more stable lubricating film over a wider temperature range.

**Figure 7:**
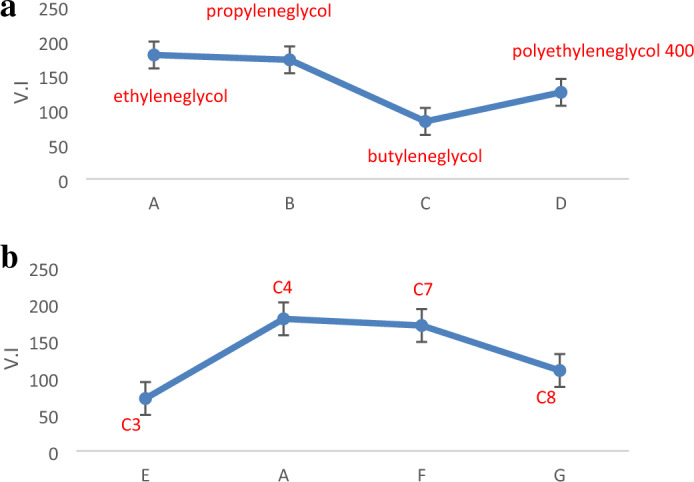
(**a**) Effect of increasing the number of carbon atoms of the used *glycols* on *viscosity index* results. (**b**) Effect of increasing the chain length of acid used in *ester* on *viscosity index* results.

The synthetic esters' viscosity was tested three times at 40 °C and 100 °C. The average values were displayed in Table 6. By raising the number of carbon atoms in the *glycols* utilised, *viscosity index* values drop, as seen in Fig. 7a. Consequently, utilising *ethylene glycol* in (A) results in superior *viscosity index* values ***VI: 179***. The *viscosity index* values in Fig. 7b show increase with increasing internal chain length of the *acid* up to *valeric acid* (5 carbons) in (A) with ***VI: 179***, then decrease with the use of *heptanoic acid* (7 carbons) with ***VI: 170*** and *octanoic acid* (8 carbons) with ***VI: 109*** because the attraction among molecules of *monoalcohols* increases as the *acid*'s carbon number increases. As a result, oils might be classified as multirange oils. Except for those prepared by *heptanoic* and *octanoic acid*, all created *esters* had *pour points* equal to or less than − 42 as a result of a rise in molecular weight. As indicated in Table 6, *density* values decrease as the chain length of the *aliphatic glycols* increases. All of the produced compounds have *densities* ranging from 0.90 to 1.06, and *flash point* range 140: 200.

**Table 6:** Physico-chemical characteristics of prepared *esters.*

Designation	The reactants for prepared dibasic ester	Pour point ASTM-D 97	The kinematic viscosity at 40°C ASTM D 445	The kinematic viscosity at 100°C ASTM D 445	Viscosity index ASTM D 22–70	Density (gm/cm^3^) ASTM D 1298	Flash point ASTM D 92
A	2 mol. *Valeric acid* + 1 mol. *ethylene glycol*	≤ − 42 °C	02.6	1.1	179	0.9800	200
B	2 mol. *Valeric acid* + 1 mol. *propylene glycol*	≤ − 32 °C	02.9	1.2	172	0.9603	187
C	2 mol. *Valeric acid* + 1 mol. *butylene glycol*	≤ − 42 °C	02.4	1.0	83	0.9575	155
D	2 mol. *Valeric acid* + 1 mol. *poly ethylene glycol (400)*	≤ − 42 °C	18.4	4.1	125	1.0601	170
E	2 mol. *propanoic acid* + 1 mol. *ethylene glycol*	≤ − 42 °C	01.5	0.7	71	1.0407	140
F	2 mol. *heptanoic acid* + 1 mol. *ethylene glycol*	− 9 °C	04.2	1.6	170	0.9547	167
G	2 mol. *octanoic acid* + 1 mol. *ethylene glycol*	− 15 °C	04.1	1.5	109	0.9745	160

## Conclusion

According to the findings of this investigation, the *ester* formed by the reaction of *valeric acid* with *ethylene glycol* as (A) exhibits better value of VI: 145 and by different *glycols* used, the VI decreases by raising the chain length of glycol used. Most of the prepared *esters* provide *pour points* from ≤ − 30 °C to ≤ − 42 °C except in case of using higher *acids* like *heptanoic acid* and *octanoic acid* in preparation the *pour point* increases to − 9 °C and − 15 °C. All of the *esters* that have been prepared are *Newtonian* fluids. It was found that the rate of biodegradability of the prepared *esters* by degrading bacterial isolates (B1, B2) in the range from 34 to 84% after 3 days of incubation period. The *ester* resulting from reaction of *valeric acid* with *ethylene glycol* give higher biodegradability with 84%. We recommend that the prepared *esters* be used as a biodegradable synthetic lubricating oil based on our findings.

## Data Availability

Data available on request from the corresponding author Reham I. El Shazly, E.mail: reham_chemidt21@yahoo.com.
